# Carl Schmitt and Democratic Backsliding

**DOI:** 10.1057/s41296-023-00625-5

**Published:** 2023-03-13

**Authors:** Ireneusz Paweł Karolewski, Xie Libin, Haig Patapan, Gábor Halmai, Acar Kutay, Petra Guasti, William E. Scheuerman

**Affiliations:** 1 Institute of Political Science, Leipzig University, Beethovenstrasse 15, 04107 Leipzig, Germany; 2 Sino-German Institute of Law/School of Institutions, China University of Political Science and Law, Xituchenglu 25, Haidian District, 100088 Beijing, People’s Republic of China; 3 School of Government and International Relations, Griffith University, Nathan Campus, Brisbane, QLD 4111 Australia; 4 Department of Law, European University Institute, Robert Schuman Centre, Villa Schifanoia, Via Giovanni Boccaccio, 121, 50133 Florence, Italy; 5 Faculty of Business Administration and Social Sciences, Molde University College, Britvegen 2, 6402 Molde, Norway; 6 Faculty of Social Sciences, Charles University, Pekarska 16, 155 00 Prague, Czech Republic; 7 Political Science and International Studies, Indiana University, 210 Woodburn Hall, Bloomington, IN 47405 USA

Carl Schmitt has been an inspiration to theorists and ideologues both on the right and on the left. On the left, for instance, Chantal Mouffe (1997) has been at pains to develop a deradicalized version of Schmitt’s friend–enemy distinction to legitimize her agonistic politics of productive and democratic conflict. On the right, conservative voices such as Adrian Vermeule use Schmitt to highlight the limitations of liberalism, proclaim the relevance of sovereignty against legality, and stress the identitarian effects of Catholic Christianity, which are explicit in Schmitt’s writings (e.g., Vermeule., 2009, 2017). Schmitt seems to be everywhere: in western capitalist democracies, former communist countries, and mixtures of the two, such as contemporary China. Even Russia was framed as Schmittian (Auer, 2015), before it openly became fascist (e.g., Motyl, 2016).

Schmittian inspirations come mainly in two forms. Some political actors might have never heard of Schmitt, let alone read his texts. They may still act in Schmittian ways, as they pursue the same way of thinking about the nature of politics and frame their own authoritarian drive perversely as the core of democracy. Therefore, Schmitt’s political theory can serve as a useful framework of analysis to better understand the political processes and political decisions in question. Other inspirations are more straightforward, as some political actors directly draw on Schmitt’s writings in their political and ideological claims and aspirations. They are expressed by scholars, propagandists, spin-doctors, and political influencers. Here, Schmitt serves as a direct source of political ideas and claims to legitimacy with regard to specific political actors and regimes.

Authoritarian populist leaders such as Donald Trump, Xi Jinping, Recep Tayyip Erdoğan, and Viktor Orbán are often described as Schmittian characters, even though they represent an entire spectrum of populist personalities: from the semi-illiterate Trump to the internationally educated Orbán. Take Trump, for example: even though he likely never heard of Schmitt, his advisors, such as Steve Bannon, had – and they gave Schmittian meaning to Trump’s political instincts. For other populist actors, the bond with Schmitt is quite overt. For instance, Jarosław Kaczyński, the powerful head of the ruling PiS party in Poland, is said to be a great fan of Schmitt’s writings – as was his doctoral supervisor at Warsaw University in the 1970s, the influential legal scholar and pre-1965 Stalinist ideologue Stanisław Ehrlich. Ehrlich postulated a ‘non-dogmatic’ approach to the rule of law and a decisionist approach to politics, in which the ‘political will’ of the leader trumps formal institutions (Zomerski, 2020). Ehrlich coined the phrase ‘the center of political disposition’ to describe the locus of real power that operates from behind the façade of a separation of powers and democratic procedures. Hence, it is often argued that Kaczyński has embraced Ehrlich’s idea as a *Leitmotif* of his political thinking (e.g., Bunikowski, 2019).

The key problem with Schmitt has always been that his writings attract both democrats concerned about the weaknesses of liberal democracy, on the one hand, and populists interested in the demolishing liberal democracy under the pretext of true democratization, on the other hand. Schmitt himself supported reactionary nationalists prior to 1933 and the Nazis after that, and he exerted a palpable influence on the intellectual life of the post-war Federal Republic of Germany. He published extensively and corresponded with leading intellectual figures in post-1945 Germany, such as the historian Reinhart Koselleck, the philosopher Hans Blumenberg, and the theologian Dietrich Braun. He also apparently influenced the thinking of Ernst-Wolfgang Böckenförde, the famous professor of constitutional law and influential judge of the Federal Constitutional Court (Müller, 2003). As Dirk van Laak (2014) shows in his brilliant book *Conversations in the Safety of Silence. Carl *Schmitt *in the Intellectual History of the Early Federal Republic*, Schmitt’s intellectual impact on the young Federal Republic was complex and certainly not marginal, despite his absence from academic institutions. He exerted much more influence on (conservative and social) democrats than other authoritarian thinkers like Julius Evola, even though Schmitt’s ideas were equally anti-pluralist, anti-liberal, and ultimately also anti-democratic. In recent years, the intellectual reception of Schmitt’s thought seems to have become increasingly apologetic. As Hasso Hofmann (2020, p. XXX) stresses in the sixth edition of his seminal work on Schmitt, *Legitimacy against Legality*, some apologists even raised the bold claim that Schmitt actually defended the Weimar Republic, while they conveniently ignored about forty of Schmitt’s publications that were pro-fascist and had antisemitic overtones.

The contributors to this Critical Exchange focus on Schmitt’s role in contemporary processes of democratic backsliding – both as a direct source of inspiration and as a framework of analysis. They identify Schmittian inspirations in countries where powerful political actors, movements, and ideologues draw on Schmitt, or act in accordance with Schmittian views, to promote attacks on liberal democracy and try to accelerate further autocratization of already authoritarian regimes. A number of Schmittian developments can be associated with democratic backsliding, a process of weakening and hollowing out the institutions of liberal democracy (e.g., Grzymala-Busse, 2008; Sata & Karolewski, 2020). This includes the infamous ‘state of exception’ as a means to usurp power, populist attacks on the rule of law and independent courts, the politics of the friend–enemy distinction to mobilize support, and acclamation as an essential means of legitimizing authoritarian decision-making. As a consequence, propagandistic terms such as ‘real democracy,’ ‘people’s democracy,’ ‘sovereign democracy,’ or ‘illiberal democracy’ are window-dressing for autocratizing policies or overtly authoritarian political regimes.

With his critique of the Weimar Republic and his ideological support for political attempts to transform it into an authoritarian presidential regime (Müller, 2003, p. 3), Schmitt delivered key concepts to legitimize such transformation. According to Schmitt, the very essence of politics is citizens’ ability to frame others as enemies rather than political opponents and, as a consequence, to become politically mobilized with the goal to support the decisions of a leader in the face of existential threat. The designation of the enemy thus establishes the political legitimacy of the leader – for Schmitt, this was the president until 1933 and afterward the Führer –  since parliamentary democracy can offer only temporary and occasional solutions to the problems of the citizenry (Schmitt, 2004 [193], p. 14). Schmitt’s claim is that only a decisive leader, who constructs the legitimacy of the state along the friend–enemy distinction, can guarantee the real equality of citizens by means of this distinction and in the face of death and conflict.

The weakening of parliaments and the empowerment of the executive is one of the key characteristics of democratic backsliding. Take as examples Turkey, Hungary, and Poland, where leaders who hoard power make decisions through their parties, networks of cronies, and captured courts in order to circumvent parliaments. They rule by decree, sideline opposition, and organize ‘national consultations’ to communicate directly with their supporters. This communication is framed as the ‘will of the people,’ while critics are silenced through imprisonment, character assassination in the government-controlled mass media, and financial pressure. This is often accompanied by myths of the political effectiveness of strong leaders, who ensure fewer problems, a better life and national greatness. Kaczyński, for instance, criticizes liberal democracy for its structural inability to solve collective problems of societies – what he calls ‘impossibilism’ – as parliaments perpetuate endless debates, and liberal elites claim legal constraints imposed by the country’s constitution or the European Union.

Schmitt diagnoses situations when the parliament is incapable of decision-making in the name of the people with a phantom of ‘parliamentary tyranny.’ Since the parliamentary majority controls the process of law-making, it has the power to legislate anything, including solutions that are opposed to the will of the people (Schmitt, 1923 [1988]. Therefore, the president can serve as a unitary and a unifying institution likely to come closer to the popular will. The president can rule using decrees, which have normative superiority through the direct legitimacy of the leader. While the inefficiency and perhaps even tyranny of the parliamentary majority poses a danger for the rule of the *demos*, the powerful leader can redeem the decaying republic. As Schmitt puts it in the conclusion to *Legality and Legitimacy:*

The people can only respond yes or no. They cannot advise, deliberate, or discuss. They cannot set norms, but can only sanction norms by consenting to draft sets of norms laid before them. Above all, they also cannot pose a question, but can only answer with yes or no to a question placed before them … In terms of its significance, the process is no longer an election, but rather a plebiscite (Schmitt, 2004 [1932], p. 90).

This supremacy of presidential power vis-à-vis parliament and citizenry is only one of several key aspects of Schmitt’s political theory which will be discussed in the contributions to this Critical Exchange. The contributors focus on several countries that exhibit Schmittian inspirations: China, Hungary, Turkey, the Visegrád countries of central and eastern Europe, and the United States. This is a selection of the more interesting cases that represent consolidated democracies, autocratic regimes, former communist countries, and autocratizing polities.

Xie Libin and Haig Patapan explore the largely favorable reception of Schmitt’s thought in China, where Schmitt influences very different groups, such as the China Path (focusing on the institutional set-up with the dominance of the Chinese Communist Party), the New Left (drawing on the Frankfurt School, highly critical of capitalism), and liberals. While China Path uses Schmitt’s concept of the sovereign to justify the unquestioned rule of the Chinese Communist Party, New Left wants to return to the Mao era of romanticized radical equality. The Schmittian state of exception here serves to justify a new wave of authoritarianism, which, in Schmittian fashion, is said to represent a better version of democracy. Libin and Patapan argue, however, that there are limitations to the application of Schmitt’s ideas in China. In particular, Schmitt’s concept of homogeneity implies an ethnonationalist understanding of the people, which poses a challenge to the current formal institutional set-up in China, based as it is on ethnic diversity. At the same time, the Schmittian notion of acclamation as an instrument of general will might be difficult to justify, unless there is a return to a Maoist cult of personality and a weakening of the Communist Party as the key political institution.

Gábor Halmai uses Schmitt’s account to trace Hungary’s road from liberalism to autocracy after FIDESZ’s electoral victory in 2010. Halmai shows how FIDESZ has carried out systematic violations of democratic standards, initiated the introduction of extraordinary powers, and played a cat-and-mouse game with the European Union regarding standards for the rule of law. Schmitt’s disdain for liberal institutions of checks and balances and judicial review found support among leading figures of FIDESZ, including the co-founder of the party László Kövér. Such disdain is also exhibited by Orbán, who has been consistent in his violation of liberal institutions, including those created after FIDESZ electoral win in 2010. For instance, Orbán violated FIDESZ’s illiberal Fundamental Law when he introduced unlimited emergency powers during the Covid-19 pandemic. Halmai also examines the ideological interplay between some current Schmittian thinkers and political developments in Hungary, as the country has become the primary European playground for democratic backsliding.

Acar Kutay explores Turkey as a case of Schmitt’s populist authoritarianism. Erdoğan appears as a Schmittian leader without direct reference to Schmitt’ writings but very much in tune with Schmitt’s understanding of politics. Kutay argues that Schmitt’s peculiar understanding of democracy can be used as a framework for analysis explaining the emergence of a populist authoritarian regime during the Justice and Development Party period and the charismatic leadership of Erdoğan. In tune with Schmitt’s ideas, Erdoğan introduced a friend–enemy distinction and identified with the masses against an allegedly corrupt and oppressive westernized elite. Moreover, he bypassed constitutional boundaries on several occasions, justifying these violations with reference to the legitimacy granted to him directly by the will of the people to rejuvenate the Turkish state. Erdoğan was also in tune with Schmittian logic as he relied on an image of a pure and homogenous people to exclude the opposition. By relying on his charismatic leadership, Erdoğan intended to establish a direct connection with the people without being restricted by core institutions of liberal democracy, such as parliament and political parties. Similar to other Schmittian autocrats, Erdoğan claimed to have established a true democracy. Kutay demonstrates how Erdoğan used the notion of a state of exception on numerous occasions to justify and consolidate his power.

Petra Guasti discusses links of contemporary populism with Schmitt’s repertoire of ideas in the Visegrád Four (V4): the Czech Republic, Hungary, Poland, and Slovakia. She focuses on the figure of the sovereign dictator and his position outside of the constitutional order, on the one hand, and on the friend–enemy distinction, on the other. In particular, Guasti shows that during the Covid-19 pandemic, sovereign dictatorship and the friend–enemy distinction were at play in all V4 countries, where populists with authoritarian leanings formed governments. Nevertheless, Guasti argues that only Hungary’s Orbán came close to embodying the Schmittian figure of sovereign dictator due to his power of decree. According to Guasti, V4 populism is fused with plebiscitarianism, in which citizens play the limited role of spectators. The region also succumbed to nativist frames of the people, portraying migrants and local ‘others’ as enemies of a homogenous people. The intensity of nativism varies across the V4, with Hungary and Slovakia constituting the pioneers of nativist tendencies. In addition, claims of misrepresentation are common in the region, reflecting Schmitt’s image of parliaments as places where various groups can take over the political institutions and sever them from the true will of the people. Guasti argues that authoritarian populism in the V4 follows Schmitt’s notion of politics based on a deeply flawed myth of the unity of a homogeneous people, which violates the very basis of liberal democracy.

In his analysis of Schmitt’s inspirations in the United States, William E. Scheuerman explores Trump as a ‘Schmittian backslider.’ Scheuerman begins by considering an internal tension with regard to Trump’s Schmittianism. On the one hand, Trump attacked independent institutions, showed disdain for democratic procedures, and used the resources of a powerful president claiming to represent the will of the nation. All this fits very well with Schmitt’s picture of a powerful leader. On the other hand, there is Trump’s administrative incompetence, his failure to grasp political complexities, and his pathological narcissism, which stand in the way of his full embrace of Schmittianism. Still, key elements of Schmitt’s thought, such as the idea of a homogeneous people or an understanding of the president as a quasi-monarch characterized Trump’s presidency. Trump treated his own activities and those of his cronies as above the law, enacted discretionary emergency powers when politically convenient, and used law against his opponents and ostensibly ‘hostile others,’ such as migrants or liberal elites. This strongly reflects the core of Schmitt’s political theory, which reframes pseudo-democratic authoritarianism as true democracy. The authoritarian tendencies of Schmitt’s thought notwithstanding, Schmitt arguably also captured a range of liberalism’s pathological political and legal tendencies that stem from contemporary capitalism. Scheuerman proposes to supplement the limited focus on Schmittian personalities with a consideration of these structural factors to fully understand the democratic backsliding.

This Critical Exchange suggests that Schmittian inspirations abound in countries that experience democratic backsliding. Sometimes, Schmitt’s ideas are used explicitly, but more often they are implicit in the actions of political elites. Schmitt’s concepts belong to the repertoire of authoritarian actors who aim to demolish, delegitimize, and ridicule liberal democracy. This suggests the need for a more critical reading of Schmitt as an ideologue of authoritarianism rather than a concerned critic of liberal democracy.

Ireneusz Paweł Karolewski

## Carl Schmitt in China

Carl Schmitt admired Mao Zedong and was intrigued with China. He would therefore be pleased that China is experiencing a case of ‘Schmitt fever’ (see Fröhlich, 2017; Marchal and Shaw, 2017; Lewis, 2022). In this contribution, we explore the reasons for Schmitt’s favorable reception in China and show how important aspects of his democratic theory will limit his influence. Our core argument is that Schmitt’s theory has been valuable due to its expansive conception of democratic sovereignty which repudiates liberalism. But other elements of his thought present obstacles to his future influence in China.

We first explore the Chinese reception of Schmitt’s thought by the China Path, New Left, and liberal scholars (see also Libin & Patapan, 2020). We then turn to how his conception of the people differs from Chinese political practice and presents an obstacle to the reception of his thought in China.

Schmitt’s thought has been especially influential for a group of scholars known as the ‘China Path’ (*Zhongguo Daolu*). The China Path refers to institutional arrangements deemed fit for China, with the ruling status of the Chinese Communist Party (CCP) as its defining characteristic. China Path scholars use, inter alia, the Schmittian concept of an absolute constitution to justify the leadership of the CCP. Thus Chen (2008, p. 494) argues that Article 2 (paragraph 1) of the Chinese Constitution, which stipulates that ‘all power in the People’s Republic of China belongs to the people,’ makes the Chinese people, as represented by the leadership of the CCP, sovereign. In Chinese, the word ‘absolute’ (*juedui*) can also mean something beyond any doubt. Consequently, by resorting to the concept of an absolute constitution, and calling the leadership of the CCP the first fundamental law of the Chinese Constitution, Chen holds that the ruling position of the CCP cannot be questioned, negating any normative examination of its position of leadership.

Schmitt’s thought has been especially influential for ‘New Left’ scholars. Unlike the Old Left, the New Left no longer refers to class struggle and the dictatorship of the proletariat – concepts that were commonplace in China up to the end of the Cultural Revolution. Instead, they draw on western critical theories such as the Frankfurt School, especially Jürgen Habermas, to fight their political battles in lectures and articles. Critical of the market economy and the capitalist system as a source of corruption and inequality, they propose to return to the Mao era, which they romanticize as the golden days of equality. The New Leftist also support Chinese nationalism, thereby embracing statism and attaching great importance to Chinese sovereignty (see Xu, 2006a, 2006b; Xia, 2010; Xiao, 2011). They use Schmitt’s theory of the exception to justify authoritarianism, his friend–enemy distinction to explain class differences, and his critique of liberalism to defend the primacy of democracy as they understand it.

New Leftists use Schmitt’s claim that the sovereign is he who decides on the state of exception in order to justify the Chinese system of government. For example, leading New Leftist Zhang Xudong (2014) reiterates Schmitt’s thesis that the sovereign may, and sometimes even must, make decisions outside the legal framework, ranging from political and economic reform to the 1989 Tiananmen incident, in order to safeguard the existence of the state. Due to the decline of the analytical purchase of the concept of class, the New Leftists turn to Schmitt’s concept of friends and enemies to account for political conflict. For example, philosopher Wang Hui (2002, p. 44) resorts to Schmitt’s diagnosis of the age of neutralizations and depoliticizations in order to revisit the concept of ‘struggle between two ways’ (*luxian douzheng*) – that is, political struggles between different ideological factions within the CCP. Finally, New Leftists criticize individual rights and liberalism as serving the interests of elites and call for greater democracy by relying on Schmitt’s repudiation of liberalism and his reconciliation of dictatorship and democracy (see, for example, Gan, 1997, 1999).

Schmitt’s attack on liberalism has forced Chinese liberals to confront and repudiate important elements of his thought in order to pursue liberal constitutionalism in China. Thus, in contrast to the China Path and New Left, notable liberals, such as Liu (1998), Qin (2000), He (2002), Mao (2006), or Ji (2007), deny the relevance of Schmitt for theoretical insights or political reforms in contemporary China. For some, such as Gao (2006), Schmitt’s writings, including those published in the 1920s before his cooperation with the Nazis, are closely linked with fascism. Therefore, they should not be regarded as philosophical works or as applicable to contemporary political debates China. Other liberals like Xu Ben (2006a; 2006b) have criticized Schmitt’s distinction between friend and enemy as a form of political theology and a call for a politics based on religious inspiration that excludes reason and potentially justifies irrational prejudices. They thus regard Schmitt’s distinction as diametrically opposed to constitutionalism. Finally, liberals have challenged Schmitt’s conception of decisionism as undermining constitutionalism and individual rights (He, 2002; Ji, 2007; Zhu, 2021).

As this overview suggests, Schmittian concepts are deployed in a variety of ways in China. There are, however, important aspects of Schmitt’s democratic theory, especially his conception of the people, and the determination of its will, that diverge from contemporary Chinese practice, demarcating lines of resistance to a comprehensive adoption of Schmitt’s political and jurisprudential ideas in China.

Schmitt (1985a, 1985b) admits that democracy is the foundation for the legitimacy of modern constitutions. But if democracy is rule of the people, how does Schmitt conceive of the people? For Schmitt, democracy assumes an existential quality of political unity based on homogeneity that points to ‘ethnonationalism’ (Salzborn, 2017, pp. 19-21). Thus, he claims that democracy ‘requires, therefore first, homogeneity, and second – if the need arises, elimination or eradication of heterogeneity’ (Schmitt, 1988, p, 9). Homogeneity can be achieved through distinctions on the basis of virtue, religion, nationality and class (Chen, 2021). It is in this context that the complex question of how to understand the concept of the people in China needs to be explored.

The Mao era drew a sharp distinction between the people and its enemies, resulting in a concept of a homogeneous people which resembles Schmitt’s conception. In Mao’s 1957 speech, later published as *On the Correct Handling of Contradictions among the People,* he distinguishes two contradictions which require different methods of resolution: the contradiction between the people and the enemy; and contradictions among the people. The first contradiction, which he calls antagonistic, requires that the state, as a people’s democratic dictatorship, suppress the enemy by various means, including the use of criminal law, disenfranchisement, and limitations to free speech. The second contradiction, among the people itself, is to be solved within the framework of democratic centralism, where citizens are permitted to enjoy democratic rights and freedoms. The distinction between these two kinds of contradiction is based on the people–enemy distinction, which was deployed extensively and ruthlessly until the end of the Mao era.

In the post-Mao era, as economic development replaced class struggle as the foremost priority of the Chinese government, the main contradiction between the people and its enemy was replaced by a tension between the people’s desire for a better life and the underdeveloped productive forces. Under these circumstances, the people–enemy distinction gave way to a more inclusive conception of the people. For example, the Preamble of the 1982 Constitution stipulates that, while the exploiting classes have been extinguished as classes, class struggle continues. Owners of businesses, previously stigmatized as exploiters, were now tolerated and increasingly supported in their initiatives. A milestone was the ratification, at the Sixteenth Party Congress in 2002, of Jiang Zemin’s ‘Theory of Three Represents’ as the guiding socio-political theory. According to this theory, the CCP supports the development of advanced productive forces, in effect allowing entrepreneurs to join the party, such that it could no longer be described as the vanguard of the working class but rather of the whole Chinese people. In this sense, the concept of the people now admits of heterogeneity.

Because China no longer differentiates between the exploiting and the working classes, Schmittian homogeneity of the people could theoretically be realized in terms of ethnicity, as the Han people constitute the overwhelming majority of the population. Yet China has rejected ‘Han chauvinism,’ recognizing fifty-six ethnic groups with distinct identities. Thus the Constitution recognizes ‘ethnic groups’ not only in its Preamble, but also in its substantive sections, for example those that reject ethnic discrimination (Art 4, para 34, 89 (11)), call for the development of ethnic administrative areas (Art 30, paras 95, 97, 99, 102, 107 and subch 6 of Ch 3) and representation of ethnic minorities in the NPC (Art 59), appoint an Ethnic Affairs Committee (Art 70), and grant the right to use ethnic languages (Art 139). Therefore, both in terms of class and ethnicity, contemporary Chinese politics makes Schmitt’s idea of a homogenous people inapplicable.

Schmitt’s defense of democracy as rule of the people raises a further question, namely how the will of the people is to be ascertained and exercised. Rejecting parliamentary or representative democracy, Schmitt’s answer is a sovereign dictatorship, where a dictator will decide on the exception in the name of the people (Schmitt, 1988). The dictator discerns the will of the people through acclamation (Schmitt, 2014, p. 112; 1988, p. 16). A similar approach was arguably pursued during the Mao era, when Mao’s cult of personality constituted a form of Schmittian acclamation. Mao's personality cult reached its highest point during the Cultural Revolution, when 1.2 billion of his portraits circulated in China, 4.8 billion Mao badges were manufactured, and everyone was expected to carry a selection of Mao’s quotes (Barmé, 1996). When Mao appeared at public gatherings, he was usually greeted by thunderous ovations, which very much reflects Schmitt’s idea of acclamation. Since Mao derived his legitimacy from his personal charisma, the National People’s Congress (NPC) was marginalized. Though starting in 1954 and convening on a yearly basis, it failed to convene for the ten-year period from 1965 to 1975. After the Mao era, Deng Xiaoping realized the harmful effects of a personality cult and began strengthening institutions. Consequently, under the present 1982 constitution, the NPC, with almost 3000 deputies elected from over 30 electoral units, is the highest organ of state power and convenes every year. Thus, the Chinese Constitution entrenches the sovereignty of the people through voting and regular elections rather than acclamation. These institutions present a formidable obstacle to the Schmittian attempt to reconcile dictatorship and democracy through acclamation.

Schmitt’s theory has been widely received in China. And indeed, his democratic theory is useful for understanding Chinese political practice in the Mao era. Yet important elements of Schmittian thought, such as his notion of the people and its will, are at odds with post-Mao era Chinese political theory and practice. Even though Schmitt will most certainly continue to be relevant in China, essential aspects of his democratic theory will limit the attempts to deploy his thought to understand and shape Chinese politics.

Xie Libin and Haig Patapan

## Hungary’s road from liberalism to illiberalism and autocracy: A Schmittian perspective

Hungary was one of the first and most successful cases of political transition after the fall of the ‘Eastern Block’ in 1989. This transition produced all necessary institutional elements of a liberal constitutional democracy, including checks and balances, guaranteed fundamental rights, and the rule of law. At the same time, Hungary is also the first and paradigmatic case of constitutional backsliding from a fully fledged liberal democratic system to, first, an illiberal and, second, autocratic regime. This contribution describes these transformations from a Schmittian perspective.

Hungary shared with other transitioning countries the need to establish an independent nation-state, a civil society, a private economy, and a democratic structure, all at the same time. The system of government introduced by the comprehensive amendment of the communist constitution assumed the presence of more than two parties in parliament and coalition-governance. At the same time, the ruling parties rejected semi- or fully presidential regimes that were implemented in many post-communist countries. They also rejected a Westminster-style two-party parliamentarism modeled on the United Kingdom. Combined with a strong judicial review process, Hungary’s new constitutional system seemed to work for more than twenty years, until FIDESZ’s overwhelming electoral victory in 2010, even though Hungary lacked a liberal democratic constitution.

After 2010, Hungary became what Prime Minister Viktor Orbán called, borrowing from Fareed Zakaria (2003), an ’illiberal democracy.’ A new constitution, called the Fundamental Law, was enacted only with the votes of the governing FIDESZ party. Depicting the results of the 2010 election as a ‘revolution of the ballot boxes,’ Orbán‘s ‘revolutionary’ intention was to eliminate all checks and balances, including the parliamentary rotation of governing parties, and institutional guarantees of fundamental rights, by dismantling the independence of the Constitutional Court and the ordinary judiciary.

This was the political context in which Hungary was hit by the Covid-19 pandemic. After the very first confirmed cases, the government used the pandemic as a pretext to claim unlimited emergency powers. But the legal presumption on which the initial emergency decree 40/2020, and the subsequent emergency statute (the Enabling Act), rest, violates Fidesz’ own illiberal constitution, the Fundamental Law of 2011, which does not provide constitutional authorization either for the decree or for the Enabling Act. (Halmai and Scheppele, 2020a; Halmai and Scheppele, 2020b).

Even before the pandemic, with FIDESZ ascent to power in 2010, the authoritarian Hungarian constitutional system could be understood by drawing on Schmitt’s critique of liberal constitutionalism and its conception of the rule of law. As Heiner Bielefeld (1996) demonstrates, Schmitt systematically argues against the liberal principle of the rule of law. Although Schmitt never used the term ’illiberal’ or ’illiberal democracy,’ he saw liberalism as resulting in an indecisive parliamentary system. Moreover, his anti-pluralism and the concept of homogeneity as a precondition of a plebiscitarian, charismatic democracy (*Führerdemokratie*) are very similar to the idea of ’illiberalism’ à la Orbán.

The Schmittian refusal of liberal democracy is mirrored in the rejection of any form of separation of powers in the current Hungarian constitutional system. As László Kövér (2019), co-founder of FIDESZ and speaker of the Hungarian Parliament, has declared, the ‘concept of checks and balances is nonsense’ and ‘the judiciary cannot be independent from the state.’

The question is whether Schmitt’s distinction between the legally bound ‘commissarial’ and legally unbound ’sovereign dictatorship’ represents a change in Schmitt’s thought, or whether it is an ad hoc response to Germany’s constitutional development from the Weimar era to Hitler’s Nazism. For Dyzenhaus (1997, p. 39), there is an essential continuity in Schmitt’s work – a diagnosis with which I agree.

It is no surprise that Schmitt’s ideas about the executive branch, as the proper locus of sovereignty, appear relevant in our own emergency situation and are used to legitimize authoritarian uses of the pandemic as a pretext for an expansion of power. For example, in an article published the day after the Hungarian Parliament’s enactment of the Enabling Act, Adrian Vermeule (2020) proposed the concept of ’substantive moral constitutionalism’ as an alternative to ’left-liberal’ constitutionalism. Vermeule prefers ‘an illiberal legalism that is not “conservative” at all, insofar as standard conservatism is content to play defensively within the procedural rules of the liberal order.’ For Vermeule liberalism is a set of purely destructive tools and procedures. The central aim of ’common-good constitutionalism’ is not to ’protect liberty’ as an end in itself, but to promote good rules and ’police power,’ which ‘despite its misleading name refers to the general power of state governments to protect health, safety, order, and public morality.’ Elsewhere, Vermeule (2018) dreams of a world in which we will ‘sear the liberal faith with hot irons’ in order ‘to defeat and capture the hearts and minds of liberal agents’ – if necessary by means of ’coercion.’

As Dyzenhaus (2020a, 2020b) rightly points out, it is a mystery why Vermeule thinks that one can have an illiberal legalism that is not ‘content to play within the procedural rules of the liberal legal order.’ Equally unclear is why Vermeule abandons the constitution altogether as an agreed basis for legal arguments. Orbán violated his own illiberal Fundamental Law when he introduced unlimited emergency powers, supervening Article 53.3 of the constitution, which limits the legal force of decrees issued in a state of emergency to fifteen days, unless Parliament approves their continuation. By enacting the Enabling Act, however, Parliament gave away this constitutional power.

To sum up, the pandemic provided a pretext for Orbán’s claims that a sovereign dictatorial power should not be controlled, even by the Parliament. For this reason, the Enabling Act – a sort of Hungarian *Ermächtigungsgesetz* – is justified in ways that echo the Schmittian defense of Hitler’s emergency measures: it is the *Führer* who protects the law. And just like during the Weimar Constitution, which was not formally abolished during the Nazi era, Orbán’s Enabling Act does not create complete lawlessness, as the constitution and laws are still in force. Nevertheless, the Nazi regime changed from an illiberal democracy into an autocracy. It remains to be seen if Hungary will follow in its footsteps.

Gábor Halmai

## Carl Schmitt’s populist authoritarianism: The case of Turkey

Carl Schmitt is a controversial author: he was the crown jurist of the Nazi regime in Germany and an antisemite. Nevertheless, it is worth engaging with Schmitt to analyze contemporary populist authoritarianism. As Lars Vinx (2021a) argues, his ideas resonate strongly in our contemporary world and are embodied in populist leaders like Trump, Orbán, and Recep Erdoğan. In previous work (Kutay, 2019), I focused on Erdoğan, adopting Schmitt’s thought in order to make sense of democratic backsliding in Turkey. I argued that Schmitt’s peculiar understanding of democracy – what I call here the Schmittian logic – casts light on the emergence of a populist authoritarian regime under the Justice and Development Party period and Erdoğan's charismatic leadership.

Of particular relevance is Schmitt’s distinction between friend and enemy, which helps us understand Erdoğan’s identification with the masses against an allegedly corrupt and oppressive elite. Also relevant is Schmitt’s account of the role of the president, which resonates with Erdoğan’s bypassing of constitutional boundaries on several occasions (Gözler, 2016). Like Schmitt’s president, Erdoğan claimed to get his legitimacy directly from the will of the nation. Erdoğan intended to rejuvenate the traditional state that was, in his mind, abrogated by western-oriented elites. However, his understanding of collective will formation is incompatible with liberal democracy. As a populist authoritarian, following the Schmittian logic, Erdoğan has relied on an image of a homogenous people and has antagonized the opposition. He has also rejected compromise and deliberation. Instead, Erdoğan has relied on his charismatic leadership in order to establish a direct link with the people without being restricted by mediating institutions, such as the parliament and political parties. While he claims to have thereby stablished true democracy, what he has actually constructed is a version of Schmitt’s democracy, which distorts (liberal) democracy for at least three reasons.

First, for Schmitt (1985a, 1985b [1932], p. 9), democracy requires homogeneity rather than pluralism. He argues that parliamentarism and democracy are built on contradictory principles: whereas parliamentarism is based on an exchange of opinions, deliberation and compromise, true democracy requires homogeneity, identification of the masses with a charismatic leader, and the elimination of mediating institutions such as political parties, interest groups and trades unions. Such an understanding of democracy restricts political participation to acclamation. There is, therefore, in Schmitt’s understanding of democracy no room for pluralism, where electoral competition is not easily differentiated from a plebiscite. Therefore, Schmitt’ understanding of democracy has distinctly authoritarian traits.

Second, Schmitt construes popular sovereignty in a way that is at odds with public autonomy, as there is no popular participation. His version of democracy relies on the leader’s acclamation by the people. This may imply a notion of consent, but acclamation is anything but a democratic practice. A political decision is not the outcome of popular participation and public deliberation but rather a mystical event in which the people decides to distinguish their enemies from their friends.

Third, Schmitt reworks key concepts in modern political theory, such as democracy, constituent power, sovereign decision, representation and legitimacy in light of the friend–enemy distinction as the underlying logic of politics (Schmitt, 2007 [1932]). For Schmitt, this is an ontological distinction which allows a collectivity to become a political community (Schmitt, 2008 [1928], pp. 257-264). The constituent power of the people is manifest in this political decision, and the president identifies with this decision (Schmitt[, 1932] 2004, pp. 67–83). The president, then, represents the political unity of the people, and this representative function explains the president’s authority to bend or suspend the law in a state of exception.

It is worth noting that Schmitt characterized the early Turkish Republic as an exemplar of his understanding of democracy (1985 [1932], p. 9). He argued that the rulers of the new regime intended to construct an ethnically homogenous society, as evidenced by a population exchange between Greeks in Turkey and Turks in Greece. His ideas can also help us make sense of the current regime’s quest for unbridled presidential rule.

Erdoğan and his political movement challenged and eradicated the secular and modernist establishment, which they claimed was preventing the expression of the nation’s will. However, the elimination of the establishment did not clear the path for liberal democracy. Even though Erdoğan and his party established civilian control over state institutions, like the judiciary, the military, and the bureaucracy, the regime also removed checks and balances, hollowed out the rule of law, and limited basic civil rights, such as freedom of speech and assembly. Echoing Schmitt’s conception of political compromise and parliamentary deliberation as obstacles to democracy, Erdoğan’s regime intended to remove all forms of political mediation between the people and the leadership. We can see this in a change of the constitution in 2017, which established a presidential system and accorded the president extraordinary powers to suspend, bend, or break positive law.

The charismatic leadership of the president is key to understanding the Schmittian logic that informs contemporary populist authoritarianism in Turkey. Schmitt insisted that the identification of ruler and ruled could only be achieved by the president. Schmitt also suggests that the president decides in and on the state of exception. This involves presidential legal authority to suspend the law. Schmitt associates such power with the commissarial dictatorship in Roman law (2013 [1921], p. 3). Just as Roman commissarial dictators returned to the constitution after restoring law, Schmitt’s president must guard the constitution when acting as a commissarial dictator. Because the actions of the president are unchecked by positive law during a state of exception, there is a risk that exceptional measures might violate human rights. For Schmitt, such violations might be necessary to restore order, even as he rejected the notion of human rights as a cosmopolitan, liberal idea. Thus, the commissarial dictator restores the constitution in the particular sense in which Schmitt understands it.

Schmitt’s account of the constitution turns on the distinction between an absolute constitution of the state and a relative constitution (Schmitt, 2008 [1928], p. 67). Whereas the former refers to the ‘soul’ that gives existence to and shapes the form of the state, the latter refers to the collection of laws that regulate the functioning of the state. Schmitt’s absolute constitution is thus a myth or ideology that embodies the identity of the state and reflects the political decision of the people (Schmitt, 1985a, 1985b [1923], ch. 3 and ch. 4). This myth or ideology grants legitimacy to the commissarial dictator’s restoration of order. Yet Schmitt (2013 [1921], p. 119) also allows for the possibility of the people giving themselves a new constitution through the sovereign decision of a dictator.

On the one hand, Schmitt’s logic requires that a new (absolute) constitution be created through a political decision and legitimated by a new ideology or myth. On the other hand, the sovereign dictator, representing the will of the people, must identify with the political decision to legitimate the foundation of the state.

Schmitt’s decisionism can help us understand the political struggles in Turkey. Whereas the political decision concerns drawing the boundaries between friend and enemy, the sovereign decision refers to deciding on whether there is an exception and what must be done to eliminate it (McCormick, 1997, p. 169)*.* In Turkey, these decisions were manifest as follows:

1. The decision on the state of exception played out in political Islamists’ and conservatives’ characterization of the republican regime as an exception. They argued that a republican system was an aberration from the authentic historical identity of the Turkish state embodied in the Ottoman and Seljuk empires. This traditional political form, which was abandoned by the new republican regime, maintained social order (*Nizam- ı Alem*) and was committed to the world domination of Islam (*i'lay-ı kelimetullah*) (Bora, 2015, p. 32; see also Ocak, 1998; Turan, 1969). Conservative intellectuals and political Islamists regard the traditional state as a metaphysical and trans-historical entity, suggesting that the Turkish state has historical continuity and eternal existence.
2. The conception of the state in this discourse recognizes the identification of rulers and ruled through religion and involves the image of an imperial state. The elites of the new Turkish Republic, however, refused to accept this legacy and adopted a less ambitious, because non-imperial, state, by aligning the interests of the Turkish state with western countries. For the political movement of Islamists and conservatives, the new regime’s deviation from the traditional essence of the Turkish state constitutes an exceptional situation.
3. The second decision, namely the decision in a state of exception, concerns the creation of a regime which reflects the true culture and identity of Turkish society. Erdoğan and his political party sought to impose a new decision and to change the values of state and society. While the Kemalist elites intended to establish a secular and modernist state by eliminating the monarchy and caliphate, and by confronting the influence of religion and tradition in society, Erdoğan seeks to reverse this decision, thus challenging the absolute constitution of the state. For example, Erdoğan’s understanding of the people conflicts with that of the new republican regime, insofar as Erdoğan positioned the people in opposition to the old regime’s modernist and secular image of the people. For Erdoğan and the political movement he stands for, the people were misguided by corrupt elites who imposed western norms and values on society. However, according to the political narrative that Erdoğan and his party have relied on, only the ‘proper people’ – as defined by the leader – can have the right to give themselves an absolute constitution.

What is more, Erdoğan successfully linked the first sovereign decision to opposition to the Kemalist establishment as a figure of enmity. Erdoğan and his party claim to represent the political unity of the nation by aligning with two discourses that challenge the hegemony of the Kemalist establishment in the state: first, a discourse that opposes an elite center to the dominated and excluded periphery; and second, a discourse that opposes the repressed Muslim majority of civil society to the privileged and oppressive bourgeois state (Küçükömer, 1969; Mardin, 1973). Accordingly, elites are said to culturally and politically repress the Muslim majority and derail the traditional state form, while corrupting the identity of the people through policies of westernization. Because corrupt elites cannot represent the nation, the nation must be represented by others – namely, Erdoğan and his party – who can identify with the people.

Erdoğan and his party display a Schmittian logic in order to match the political subject, which holds power and capacity to determine a friend–enemy distinction, and acts as the state-building agents, who hold legitimate authority to establish a new legal and political order. The political movement under the leadership of Erdoğan has identified with the decision denominating the republican period as an aberration. It has the intention to give a political form to this decision by creating a new regime.

Acar Kutay

## Carl Schmitt and contemporary populism in the Visegrád Four

In *The Concept of the Political* (2008), Schmitt introduced two important concepts that resonate in the contemporary era of populism in the Visegrád Four (V4): the Czech Republic, Hungary, Poland, and Slovakia. These concepts are the friend–enemy distinction and the sovereign dictator. Schmitt reduces the political to the binary friend–enemy distinction along identarian axes. In Schmitt's definition, the sovereign dictator is outside of the constitutional order and formal authorization mechanisms (e.g., elections), legitimizing his rule by claiming to exercise the constituent power of the people. Schmitt's sovereign dictator is in a liminal space between an old order that has been dismantled and a new order he must constitute.

This concept of sovereign dictatorship helps us to understand the V4, where populists were in power during the pandemic: Andrej Babiš in the Czech Republic (voted out of office in October 2022), Viktor Orbán in Hungary (re-elected for the fourth consecutive term in April 2022), Jarosław Kaczyński in Poland (as leader of the Law and Justice party since 2003 and Deputy Prime Minister between October 2020 and June 2022), and Igor Matovič in Slovakia (Leader of the Ordinary People party since 2010, Prime Minister between March 2020 and April 2021, since 2021 as Minister of Finance). With varying intensity and degrees of success, each of the V4 populist leaders sought to avoid accountability and to accumulate power. However, only Orbán came close to the figure of a sovereign dictator by introducing rule by decree in the early stages of the pandemic (cf. Halmai’s contribution to this Critical Exchange, Guasti, 2020a, 2020b).

Schmitt develops the second relevant concept – that of the friend–enemy distinction – with regard to the people, which pre-exists constitutional order and materializes in the public distinction between friend and enemy. Schmitt's concept of enmity is an existential one: what binds people is their willingness to die for their own and to kill others. Because this distinction is construed in ethnic terms, Schmitt rejects the liberal notion of citizenship and argues, instead, that a state can only be ethnonationalist. The political community that constitutes the nation must, therefore, suppress, expel, or eliminate internal enemies.

Populism research focuses on constructing the ambiguous category of ‘the people’ (Canovan, 1984, 1999; Katsambekis, 2020). In her influential work, Margaret Canovan (1984) proposed three discursive frames of 'the people.’ First, ‘the people as a nation’ stresses common roots and traditions and is characterized by hostility to factions, especially the representation of particular classes or groups. Second, ‘the people as underdogs’ is profoundly hostile to hierarchy and resentful of experts and intellectuals. Third, ‘the people as ordinary man’ emphasizes the importance of the people, rather than institutions, norms and values. This notion of the people is conformist, opposed to expanding liberal rights, and hostile to impersonal economic forces. It is Canovan’s notion of ‘the people as a nation’ which captures Schmitt’s notion of the people.

Populist politics is about drawing the boundaries of who belongs to 'the people’ (Canovan, 2005) as well as delineating political demands and policies as legitimate or illegitimate (Laclau, 1977). But the construction of 'the people’ is only the first step of populism (Laclau, 2005). Even more important is the populist claim to represent the people and to make a stab at both knowing and representing the popular will (Caramani, 2017). Here, populism echoes Schmitt’s view of the leader as the embodiment of the people.

If the populist leader embodies the people, he is essentially out of control – he cannot be held accountable by the people. In fact, there can be no accountability. Populism essentially fuses with plebiscitarianism or audience democracy. Plebiscitarianism promises to ‘restore the concept of the people as a meaningful collective identity,’ while turning passive citizens into spectators (Urbinati, 2014, p. 171; cf. Sata & Karolewski, 2020). Together, populism and plebiscitarianism disfigure democracy (Guasti, 2020a, 2020b) by deploying enmity to eliminate diversity from the public forum, to expel the other, and to create a homogeneous 'people'. The populist leader dismantles accountability mechanisms, such as checks and balances and free and fair elections. Power is centralized in the leader, and the opposition is designated as the enemy of the people (cf. Ruth-Lovell et al., 2019). Plebiscitarianism also eliminates horizontal and vertical accountability by withdrawing institutions from participative and deliberative procedures (cf. Urbinati, 2014).

Across the V4, the question of (national) sovereignty has been a salient issue since the fall of the Iron Curtain, fuelling the rise of populism in the last decade (Bustikova & Guasti, 2017). The nativist frame of the *people* as a nation has been present in all four countries, especially since the 2015 migrant 'crisis.’ This trope is directed against migrants as well as ethnic minorities, including, but not limited to, Roma. The intensity of nativism among the V4 leaders varies across countries and time; it is most intense in Hungary and least intense in the Czech Republic. Another manifestation of nativism is antisemitism, especially among the populist radical right. It has been perhaps most intense in Hungary and Slovakia and almost non-existent in the Czech Republic.

The frame of the *people* as underdog is less intense across the V4, where social -democratic parties have been struggling to maintain electoral support as their voters switch to populist competitors. This is perhaps most pronounced in the Czech Republic, where the Action of Dissatisfied Citizens (ANO), the populist party founded by Babiš, 'lured away voters from Social Democrats and Communists, neither of whom crossed the parliamentary threshold in the 2021 parliamentary elections. Babiš attracted those voters through excessive welfare spending, including an increase in the minimum wage, a significant increase in pensions, and free public transport. This came at a cost: during the first three years of the Covid-19 pandemic, the Babiš government implemented the highest increase in public debt among EU member states. Similarly, the Law and Justice party (PiS) in Poland attracted voters by introducing welfare measures such as child benefits. In Slovakia, Matovič is seeking to maintain crumbling support for his party through generous welfare spending beyond the country’s means – a program that is being slowed down by his coalition partners. Orbán, the leader least prone to big welfare spending, also introduced measures such as state-guaranteed loans to young families.

The ordinary people frame is most present in the Czech Republic and Slovakia. Both Matovič and Babiš use this frame to transcend the boundaries of the left and the right in order to attract voters from both sides of the political spectrum, with Matovič even calling his party the Ordinary People party. This is an effective electoral strategy, as populist voters tend to be less interested in coherent policy or fiscal responsibility than in direct transfers. Nevertheless, it complicates governance, as evidenced by Matovič and Babiš seeking to maintain popular support once in power – an effort that resulted in erratic and irresponsible policy. For example, Matovič imported the Russian Covid-19 vaccine Sputnik V without the consent of his coalition partners. When the Slovak health agency refused to approve the vaccine, Matovič relied on Hungary to provide approval. As a result, only five thousand Slovaks were administered Sputnik V, and the rest of the vaccines were sold back to Russia. Matovič was forced to resign as Prime Minister, but he remains in full control as the party leader and became Minister of Finance (cf. Guasti and Bilek, 2022).

While much attention has been paid to 'the people’ and the relationship between populists and the people (Canovan, 1984, 1999; Urbinati, 2014), the notion of the 'elite' remains undertheorized (Bustikova and Guasti, 2019). Katsambekis (2020) criticizes the homogeneity and morality theses that are central to the ideational approach, as limiting in ‘assessing its antagonistic relationship to its “other” and thus the possible impact on democracy’ (Katsambekis, 2020, p. 16). Populist parties themselves tend to rhetorically equate the elite with the establishment, criticizing elites selectively and strategically, and constructing current power-holders, established political parties, cultural elites and civil society as the 'political establishment' (Bustikova and Guasti, 2019).

Claims of misrepresentation are at the core of populism (cf. Guasti & Almeida, 2019). In claims of misrepresentation, populists seek to establish an antagonistic relationship between 'the elite' and 'the people' and to articulate grievances using the language of the people (Canovan, 1984, 1999). Within the strategies and style of populist parties, claims of misrepresentation create space for a new insurgent 'representative' of a newly defined 'people' (on the 'chameleonic' nature of 'the people', see Taggart, 2002). Because populist claims of misrepresentation decry establishment representatives as dysfunctional, such claims are anti-establishment by nature: they attempt to persuade the constituency to turn against established actors (cf. Moffitt, 2016; Urbinati, 2019a, 2019b). Accusing elected representatives or other advocacy groups of misrepresenting 'the people’ is, therefore, a precursor to populist claims to representation (Guasti & Almeida, 2019).

V4 populists exemplify the importance of claims of misrepresentation for populist, anti-establishment parties. They define 'the people’ as those 'left behind’ by 'the elite', thereby defining the people as nation, underdog, and ordinary as well as 'abandoned' by establishment parties – what Babiš calls ‘the post-1989 cartel' (Babis, 2017).

For nativists, the people share identity, culture, interests, and self-determination. In the V4, refugees are excluded from the people, and while foreigners are expected to assimilate, it is questionable whether they can ever truly belong. More likely is their relegation to second-class citizenship or exploitation as cheap migrant labor. Moreover, there is another group that is increasingly excluded from the people in the V4, namely LGBTQ individuals and women. In all four countries, populists sought to limit the expansion of LGBTQ rights: the Czech Republic slow-walked the marriage equality act; Slovakia held a failed referendum to codify marriage as a union between a man and a woman. They also introduced specific legislation against transgender citizens and women: Hungary enforces compulsory gender at birth; Poland introduced a near-absolute abortion ban; Slovakia and Hungary are seeking to make abortion more difficult to access (Guasti and Bustikova, forthcoming).

Populism in power erodes accountability (see Ruth, 2018 on Latin America, Guasti, 2020a, 2020b on Central Europe) through the centralization of power in the hands of the populist leader (cf. Bermeo, 2016). Populism also contributes to democratic erosion on the institutional level by undermining electoral competition, judicial independence, and legislative oversight. It further constrains the people’s agency and the public sphere through an erosion of civil liberties such as freedom of association, the right to protest, and freedom of the press, which are all key to holding leaders accountable. Attacks on these cornerstones of liberal democracy serve the sole purpose of accumulating unaccountable power in the hands of the populist leader. Only the strong democratic guard rails can protect democracy from the populist leader and Schmitt's dictator.

The case of Orbán's erosion of Hungarian democracy into competitive authoritarianism exemplifies the mechanism of executive aggrandizement. Here a populist leader won free and fair elections and consolidated a constitutional majority by weaponizing conservative civil society and the media to rally people against external (refugees, the EU) and internal enemies (the opposition, civil society, ethnic and sexual minorities) (Greskovits, 2020). As the purported embodiment of the people’s will, he then dismantled the 'old' constitutional order and changed the constitution. Next, he subdued the judiciary, banned independent media, and prosecuted members of the opposition. During the Covid-19 pandemic, he also destroyed what was left of an already toothless process of legislative oversight. As a result, Orbán holds all power and rules by decree, making every subsequent election less free and unfair (cf. Sata & Karolewski, 2020).

Yet the incomplete character of the representation in the figuration of 'constituencies' and 'the people' opens up spaces for the democratic contest (Saward, 2017; Rosanvallon, 2008). The cacophony of claims of representation and misrepresentation can be understood as a contestation between competing actors seeking (democratic) legitimacy for their claims by constructing 'the people' – as the represented, and 'the elite' – as the establishment who misrepresents 'the people, thus questioning the legitimacy of established actors and posing as legitimate representatives (Disch, 2008, p. 52; De Wilde, 2013). Both representation and misrepresentation are strategically exploited by populist actors seeking to discredit their opponents, the political system, and representative democracy (cf. Caramani, 2017). In their struggle for political power, new political actors (over)emphasize the void between representatives and the represented. Claims of (mis)representation present political demands while reaffirming or challenging the legitimacy and authority of the elected representatives regarding what they do (policies), how they do it (politics), and for whom they do it (polity). Misrepresentation is a (constructed) mismatch between the existing political system and (new) political demands (Ankersmit, 2002).

Schmitt's ethnonationalist, exclusionary notion of the people, and his ideal of an unconstrained leader, have lasting allure for populist opponents of representative democracy on both the right and the left. For Schmitt, as for these leaders, democracy is merely a set of procedure for selecting a leader who embodies the people – not a system that constrains them and holds them accountable. Populist actors in the V4 embrace this Schmittian notion of democracy, along with a deeply flawed myth of the unity of a homogeneous 'people'. Because people carry multiple identities and interests, however, Schmitt’s dictator and contemporary populists are faced with a constant need to eliminate differences by an ever-narrower notion of the people which excludes and ultimately eliminates all minority groups. In this way, populism disfigures liberal democracy before eliminating it (Urbinati, 2014). Orbán has perhaps made the most progress on his way to eliminating democracy in the V4, while Babiš and Matovič have been less successful. Poland appears closer to Hungary, but it is the next parliamentary election that will decide whether Poland will follow Hungary on the road to competitive authoritarianism or end up, like the Czech Republic and Slovakia, with a struggling but still liberal democracy.

Petra Guasti

## Trump the Schmittian backslider?

It only took a few weeks following Trump’s surprising 2016 electoral victory for political commentators to unleash a cottage industry devoted to Schmitt and democratic backsliding. Schmitt’s name not only quickly appeared alongside Trump’s in the usual academic journals and edited volumes, but also in the pages of *The Atlantic*, *National Review, New York Review*, *New York Times*, and on the webpages of widely read political blogs (e.g., Lawfare). Controversies about the 9/11 terrorist attacks had already made Schmitt a household name among U.S. jurists and political theorists. Trump’s ascent took things to a new level by transforming Schmitt into a reference-point for many journalists and political writers.

The otherwise insightful ensuing debate has suffered from an internal tension. On the one hand, writers have been eager to attribute ‘Schmittian’ attributes to Trump as a political figure and personality. The center-left Brookings Institute’s Quinta Jurecic helped get this ball rolling in December 2016 by predicting that Trump would soon become the ‘first Schmittian president,’ a position she subsequently modified in light of Trump’s obvious administrative ineptness (Jurecic, 2016). By the final year of his presidency, she and her co-author, Benjamin Wittes, would instead describe Trump as a ‘fair weather’ and perhaps ‘lazy’ or ‘wannabe Schmittian, a Schmitt when it is easy,’ an assessment that overlapped with the conservative *National Review*’s claim that Trump’s personal foibles – in particular, his obvious narcissism – luckily obstructed his full-on embrace of Schmitt (Jurecic and Wittes, 2020; Sibarium, 2020).

This first line of inquiry risks personalizing the complexities of democratic backsliding and its possible ties to Schmitt’s thinking. It tends to downplay structural or systemic features of Trumpism as both a political movement and a mode of governance with some identifiably Schmittian traits. It also invites the counterargument that neither the barely literate Trump nor the semi-literates around him – with the possible exception of one of his policy advisers, the xenophobic Stephen Miller – could distinguish between Schmitt and the great émigré historian Carl Schorske. To be sure, some wannabe Trump palace intellectuals, typically based at the Claremont Institute, were, in fact, versed in twentieth-century German thought. But their philosophical guru was Leo Strauss, not Schmitt (Field, 2021). And even if some of them, like John Eastman, loomed large in Trump’s disastrous decision to challenge the November 2020 electoral results, it remains unclear that they influenced many of Trump’s policies.

On the other hand, a second, less personalized line of inquiry identifies elective affinities between Schmitt’s political vision and the broader political and institutional logic of Trumpism, specifically, and authoritarian populism, generally (Dyzenhaus, 2020a, 2020b; Mohamed, 2018; Scheuerman, 2019, 2021; Urbinati, 2019a, 2019b; Vinx, 2021a, 2021b). I limit myself here to recalling some of its main observations.

With echoes of Schmitt’s truncated view of democracy, Trumpism jettisoned an unavoidably messy, pluralistic, liberal and democratic notion of the ‘people’ for an exclusionary, homogeneous collectivity (i.e., Trump’s ‘real Americans’), viewed as partaking in a deeply shared identity and waging existential battle against internal and external ‘foes’ (immigrants, Black Lives Matter activists, ‘globalists,’ etc.). Democracy, in short, became authoritarian right-wing identity politics predicated on preserving, whenever possible, equality understood as the body politics’ substantial ‘sameness,’ usually interpreted in ethnic and racial terms. Not surprisingly, core elements of Schmitt’s autocratic hyper-presidentialism simultaneously reappeared. In the Trumpist imaginary, the single person of the quasi-monarchical president possesses a mystical link to ‘real’ (read: white) Americans, the proper sovereign body, in a fashion akin to Schmitt’s imagined ties between the popular constitution-making (or constituent) power and the Weimar President (Schmitt, 2015 [1931]). As the constituent power’s most direct institutional expression, the charismatic executive necessarily possesses vast and potentially unchecked powers.

Predictably, while in office Trump treated his own activities – and those of his allies and cronies – as above or beyond the law. Flagrant corruption and conflicts of interest within his administration were pushed aside. When politically convenient – for example, in battles with Congress over immigration and the so-called ‘border emergency’ – his discretionary emergency authority was declared to be effectively unlimited. Even when Trump’s Attorney General, William Barr, paid lip service to the ‘rule of law,’ it usually meant deploying law as a political weapon against hostile ‘others’ (e.g., immigrants, the ‘liberal elite’). Barr’s authoritarian and extreme right-wing Catholic views about law, at any rate, include some eerily Schmittian traits (Shaw, 2020).

As Lars Vinx has insightfully pointed out, Schmitt’s political theory offers a disturbingly ‘sophisticated re-description of the democratic ideal that puts that ideal into the service of’ pseudo-democratic authoritarianism (Vinx, 2021a, 2021b, p. 176). Writing prior to the violent 6 January 2021, attack on the U.S. Capitol, Vinx zeroed in on Schmitt’s interpretation of mass acclamation as a superior, authentically political mechanism to legitimize a top-down presidential regime. As an open or ‘public manifestation of the recognition of [the leader’s] charisma,’ acclamation does not require ‘free and fair’ political competition or open elections. For the charismatic leader, as for a religious prophet, the followers’ free and equal political consent is simply unnecessary. Instead, what counts is public acknowledgment of the leader’s privileged, implicitly superhuman status. Acclamation on this model ‘is something like a public confession of one’s faith’ that helps strengthen the disciples’ shared convictions. For Schmitt, acclamation can take the form of flawed or even rigged elections, ‘abused for acclamatory purposes, a popular referendum, or some spontaneous commotion on the streets'. Why? Like the religious prophet, the charismatic leader ‘will be unable to exercise effective leadership over a group of people unless his or her claim to be the embodiment of a higher power is in fact recognized’ (Vinx, 2021a, 2021b, p. 174). Yet the leader’s claim to rule is never strictly conditional on free and equal votes among ‘mere’ mortals, or a victory in elections that meet liberal and democratic criteria.

Vinx’s remarks provide a useful basis for making sense of the failed 6 January insurrection and, more generally, the Trumpists’ astonishing disdain for ordinary electoral mechanisms. Two years after the insurrection, self-described ‘America First’ political activists, who still deny the 2020 election results and tout wacko conspiracy theories, continue to gain ground in the Republican Party (Berzon, 2022). Many of them will likely oversee upcoming electoral contests. Needless to say, this is an ominous development that bodes badly for a beleaguered U.S. democracy.

Here I instead want to focus on an issue that tends to get neglected in the debate. Both those who view Trump as a Schmittian political personality, and those for whom Trumpism as a movement and mode of governance exhibit Schmittian traits, say little about the sources of Schmitt’s apparent prescience, in part because the evidence for any direct or even indirect Schmittian intellectual ‘influence’ is sparse. So how then can we explain that a controversial twentieth-century authoritarian German thinker helps decipher disturbing features of contemporary democratic backsliding? One answer has already been anticipated by Vinx: as a devotee of right-wing, mass-based authoritarianism, Schmitt grasped its key components; unsurprisingly, much of what he had to say seems pertinent to contemporary authoritarian populism. But can we say more? By posing this question, I do not intend to celebrate Schmitt’s alleged brilliance, or endorse his claim to ‘classical’ status alongside Hobbes, Weber and so on. Rather, I simply note that Schmitt’s theory captures core features of democratic backsliding; this fact requires explanation.

Similar questions were faced and answered, with mixed results, by one of Schmitt’s most astute leftist contemporaries and critics, the Frankfurt School political and legal theorist Franz L. Neumann, another mid-century figure who has occasionally re-emerged in debates about Trump and authoritarian populism (Connolly, 2017: 8; Fuchs, 2018). Deeply skeptical of Schmitt’s political agenda, yet cognizant of his theory’s diagnostic merits, Neumann defended a neo-Marxist reading of Schmitt as an ideologue of recent capitalist transformation and its accompanying political and legal tendencies. As Neumann pointedly asserted, Schmitt’s *Situations-Jurisprudenz* – that is, his embrace of exceptional or situational top-down executive rule – was best interpreted as the ‘legal theory and legal practice of bourgeois society’ as it transitioned from classical or competitive to a more organized, monopolistic phase (Neumann, 1986 [1936], p. 6). The latter phase, Neumann tirelessly documented, increasingly relied on non-general, non-traditional forms of law incongruent with the liberal rule of law, as conventionally interpreted. Core economic shifts, in conjunction with closely related political-institutional and legal developments, tended to corroborate precisely that authoritarian *Situations-Jurisprudenz* which Schmitt’s theory celebrated. The ‘crisis of parliamentarism’ which Schmitt diagnosed (1985 [1926]), alongside the authoritarian presidentialism he favored, meshed disturbingly well with real-life empirical trends whose roots ultimately lay in monopoly capitalism and its dependence on individual measures and executive decrees.

In sum, Schmitt’s theory reproduced pernicious structural tendencies operative within capitalist society: his was a right-wing theory of contemporary capitalist society’s crisis features. When critically reinterpreted and its normative aims demoted, it might provide observers, who otherwise rejected Schmitt’s own preferences, with some useful insights. To be sure, any critical redeployment of Schmitt would need to pay proper attention to fundamental shifts within capitalism to which political and legal changes were necessarily linked. In part because Schmitt lacked a sufficiently critical-minded social theory of capitalism, his views remained ideological, in the old-fashioned Marxist sense. Yet they nonetheless captured some of contemporary society’s pathological political and legal tendencies.

Not surprisingly, Neumann was a keen reader of Schmitt; he regularly tried to respond to him. Many readers of this journal will chafe at his Marxism and worry that it distorts important features of the story. What nonetheless remains relevant is Neumann’s attempt to make sense of Schmitt’s occasional diagnostic merits with recourse to a broader critical social and political theory of crisis. Clearly, any such theory will need to focus on deleterious political-institutional trends and their sometimes relatively independent political sources. Much of the existing literature on democratic backsliding or regression does so. When read against the grain, Schmitt can sometimes help to outline such a theory. But as Neumann intimated, a critical theory will additionally need to link the analysis of such political trends, in an appropriately nuanced way, to social theory and, even more specifically, to a critical account of contemporary capitalism. Such an approach will also need to reject Schmitt’s knee-jerk hostility to liberal democracy and the rule of law (Scheuerman, 1994).

Jan-Werner Müller is right to describe economistic interpretations of contemporary populism that are ‘fixated on certain classes’ (e.g., economic globalization’s postindustrial ‘losers’) as analytic ‘dead ends’ (Müller, 2016, p. 10). But that hardly justifies Müller’s and many other recent political scientists’ tendency to downplay and sometimes simply ignore complicated matters of political economy. As Neumann would have rightly responded, a critical-minded analysis of capitalism’s most recent crises has to constitute a core element of any serious discussion of present-day democratic backsliding. To deny some causality to the 2008 global financial crisis (or more recent Euro-crisis) or, more generally, dramatically heightened inequality and the rise of post-Fordist capitalism, just does not make much sense. As Philip Manow (2018), in a related vein, has suggested, variations between and among populist movements can partly be explained by the nationally divergent political-economic formations in which they arise.

Of course, we will need a serious debate about how best to consider material versus other more strictly political factors. Absent that conversation, any theory of democratic backsliding will potentially prove incomplete and even one-sided. Writers then risk reproducing Schmitt’s analytic blind spots and silences in a way that will likely distort their analyses. They no longer would have the necessary critical distance from Schmitt but instead might inadvertently integrate some of his theory’s many flaws.

William E. Scheuerman

## Data Availability

The data supporting the findings of this article are available within the article. No additional data have been used petra guasti’s work on this output was supported by the NPO “Systemic Risk Institute” number LX22NPO5101, funded by European Union - Next Generation EU (Ministry of Education, Youth and Sports, NPO: EXCELES).
